# Annotations

**Published:** 1901-12-07

**Authors:** 


### Annotations.

### Food Preservatives.

The report of the Departmental Committee of the Local Government Board, appointed a couple of years ago to investigate the question of food preservatives, cannot be called an altogether satisfactory document. In regard to milk, which is of all articles of food the most difficult of preservation, the most susceptible to change in hot weather, and the article of all others the supply of which is limited by difficulties of carriage and of preservation, they recommend that the addition of preservatives or colouring agents shall be an offence under the Food and Drugs Acts ; and this is as it should be. But surely if it is possible in regard to milk it is possible all round. The question of the addition of artificial preservatives to food does not appeal to us as one to be decided solely by the immediately poisonous effect of such preservatives upon the consumer. It is a matter of false pretence. When annatto is added to milk to give it a rich and creamy colour, and when boracic acid is added to it to prevent those changes taking place which would at once declare its age, and when such milk is sold as fresh milk, the transaction is of the nature of a fraud, however little it may be possible to prove that the particular dose of boracic acid was the cause of any particular ailment in those that took it. In regard to milk this fraud is to be put an end to, which is good, but in regard to other articles, if the recommendations of the committee are carried into effect, it will be allowed to continue, although some check is to be put upon the extent to which it may be carried. Let us, however, be thankful for such small mercies as are granted us. When Dutch shrimps, which as we are told can be kept "for months" by the addition of borax, are sold as "potted shrimps," a delicacy the freshness of which is generally held to be guaranteed by its odour ; when margarine is made to look like luscious country butter ; and when marmalade sold as "home made" is found to be pickled in salycilic acid, let us remember to look at the label, and if we are particular and have a weakness for honest food let us ask for things made up for infants and invalids, for the committee does recommend certain small concessions to honesty ; one that when any preservative is used its nature and amount shall be "notified," and the other that in the case of dietetic preparations intended for infants or invalids the use of chemical preparations shall be totally prohibited. Let us be children once again and eat real jam !

### The Notification of Small-pox.

The Mayor of Poplar has expressed the opinion that if the medical men practising in London had shown themselves more capable of diagnosing small-pox the disease would never have spread to its present extent. Perhaps it would be more correct to say that if the law had given medical men proper protection in notifying doubtful cases the epidemic would have been more easily checked. The diagnosis of small-pox, especially in its "modified" form, is full of pit-falls. Yet the law takes no cognisance of and makes no provision for doubtful cases of any kind. Thus the doctor is left in a great strait. If he notifies before he knows, that is, before he has proper and sufficient proof of the nature of the disease, he is liable to be cast in damages by the patient for the injury done him by such "negligence"; while if he delays his notification until the period of doubt is past and he really does know, or in the words of the Act becomes "aware" of the nature of the disease, then he runs the risk of prosecution by the sanitary authority for neglecting to notify. Not that the sanitary authority will get much by its motion, unless it can show that the doctor, during the doubtful period was really "aware" of the nature of the disease, and here comes in the irony of the situation. If the doctor be a wise man, far seeing, and careful of his own interests, he probably keeps his mouth shut, and awaits events. If, however, this good man be a conscientious person, he will take precautions on the chance of his patient being infectious, even though he cannot at that moment feel quite sure about it, and thereby he will show his unwisdom, for every one of these precautions will be good evidence against him when the sanitary authority, full of that delightfully easy knowledge which comes after the event, prosecutes him for delay in sending in his notification. The trouble arises not entirely from mere difficulty of diagnosis, but from the facts that in some cases positive diagnosis is impossible ; that there is nowhere to receive these doubtful cases until they clear up, and that until this happens there is no law under which they can be removed. It is all very fine to blame the doctors, but the fault lies in the law.

### The Man in the Crystal Urn.

The very curious exhibition of the man in the "crystal urn" which is now going on at the Royal Aquarium, is on a somewhat different footing from those of the various fasting men and fasting women that we have become so accustomed to of late. Papuss is a South American, aged 34, who, after being wrapped up in 400 yards of flannel bandage, has been placed in a glass box or "crystal urn," which has then been sealed up water tight, and sunk under water, where we suppose it now lies. The man, meanwhile, is supplied with air by means of a tube through which it is driven by an electric fan, but with nothing else, neither food nor water, and there he is to lie for the whole week. That this is a performance demanding very considerable endurance and fortitude no one will deny, even though the man in the urn be assisted, as he claims to be, by his power of sending himself into a cataleptic trance, and by auto-suggestion as to the unreality of hunger and the non-necessity of food. What is of some scientific interest, however, is the statement that by aid of the careful and rather tight bandaging the circulation can be so limited as to exercise a considerable influence upon the tissue waste and presumably, therefore, on the necessity for water for excretory purposes. We know, of course, that in hibernating animals the circulation goes on in a very modified way, being reduced almost to zero, probably in consequence of an influence exerted through the vaso-motor nerves, and if it could be shown that a somewhat similar though only local limitation of vital changes can be effected by external pressure, the matter would be one of very considerable interest. It will be recollected that attempts have been made from time to time, with more or less success—generally less—to control inflammatory processes by this means.

